# Effects of Low Molecular Weight Yeast β-Glucan on Antioxidant and Immunological Activities in Mice

**DOI:** 10.3390/ijms160921575

**Published:** 2015-09-08

**Authors:** Na Lei, Mi Wang, Lifang Zhang, Sui Xiao, Chengzhong Fei, Xiaoyang Wang, Keyu Zhang, Wenli Zheng, Chunmei Wang, Ruile Yang, Feiqun Xue

**Affiliations:** Department of Pharmacy, Shanghai Veterinary Research Institute, Chinese Academy of Agricultural Sciences, Shanghai 200241, China; E-Mails: aoleina@126.com (N.L.); twinzhang@shvri.ac.cn (L.Z.); xiaosui@shvri.ac.cn (S.X.); aries@shvri.ac.cn (C.F.); wxy@shvri.ac.cn (X.W.); zcole@shvri.ac.cn (K.Z.); wenli@shvri.ac.cn (W.Z.); wangchunmei@shvri.ac.cn (C.W.); yangruile@shvri.ac.cn (R.Y.)

**Keywords:** sulfated glucan, *Saccharomyces cerevisiae*, antioxidant activity, immune activity

## Abstract

To evaluate the antioxidant and immune effects of low molecular yeast β-glucan on mice, three sulfated glucans from *Saccharomyces cerevisiae* (sGSCs) with different molecular weight (*M*_W_) and degrees of sulfation (DS) were prepared. The structures of the sGSCs were analyzed through high performance liquid chromatography-gel permeation chromatography (HPLC-GPC) and Fourier transform infrared spectroscopy (FTIR). sGSC1, sGSC2, and sGSC3 had *M*_W_ of 12.9, 16.5 and 19.2 kDa, respectively, and DS of 0.16, 0.24 and 0.27, respectively. *In vitro* and *in vivo* experiments were conducted to evaluate the antioxidant and immunological activities of the sGSCs. *In vitro* experiment, the reactive oxygen species (ROS) scavenging activities were determined. *In vivo* experiment, 50 male BALB/c mice were divided into five groups. The sGSC1, sGSC2 and sGSC3 treatment groups received the corresponding sGSCs at 50 mg/kg/day each. The GSC (glucans from *Saccharomyces cerevisiae*) treatment group received 50 mg/kg/day GSC. The normal control group received equal volume of physiological saline solution. All treatments were administered intragastrically for 14 day. Results showed that sGSC1, sGSC2 and sGSC3 can scavenge 1,1-diphenyl-2-picryl-hydrazyl (DPPH), superoxide, and hydroxyl radicals *in vitro*. The strength of the radical scavenging effects of the sGSCs was in the order of sGSC1 > sGSC2 > sGSC3. Oral administration of sGSC1 significantly improved serum catalase (CAT) and glutathione peroxidase (GSH-Px) activities and decreased malondialdehyde (MDA) level in mice. sGSC1 significantly improved the spleen and thymus indexes and the lymphocyte proliferation, effectively enhanced the percentage of CD4^+^ T cells, decreased the percentage of CD8^+^ T cells, and elevated the CD4^+^/CD8^+^ ratio. sGSC1 significantly promoted the secretion of IL-2 and IFN-γ. These results indicate that sGSC1 with low *M*_W_ and DS has better antioxidant and immunological activities than the other sGSCs, and sGSC1 could be used as a new antioxidant and immune-enhancing agent.

## 1. Introduction

β-Glucans are polysaccharides of d-glucose monomers linked through β-glycosidic bonds, isolated from various natural sources including yeast, mushrooms, bacteria, algae, barley and oat [1]. β-Glucans have attracted attention over the years because of their physical and chemical characteristics, which exhibits a broad spectrum of biological and medicinal activities including anti-tumor, immune-modulating, wound healing, hematopoiesis-stimulating, anti-oxidant and anti-inflammatory properties [2,3,4,5].

β-Glucan from different sources and with different molecular weights (*M*_W_) has different activities [6]. Many studies reveal that the biological properties of various biopolymers depend on their molecular weight. The high molecular weight of glucan caused some problems such as high viscosity and low permeability into cell. However, low molecular weight polysaccharide seems to play an important role in the exploration of natural antioxidants in food industry and pharmaceuticals. *M*_W_ of β-glucan is strictly associated with its physiological effects [7,8,9]. The range of relative *M*_W_ of β-glucans is quite wide and fluctuates (depending on origin) from tens to thousands of kilodaltons (kDa) [10]. Previous studies suggested the influence of *M*_W_ of oat and barley β-glucan on diet induced thermogenesis, diabetes and cardiovascular disease markers [9,11,12]. Low molecular weight oat β-glucan effectively decreased oxidative stress parameters [13], and had strong anti-tumor properties with no toxicity for normal cells [14]. The glucan with *M*_W_ of approximately 5 kDa had the highest ability to induce IL-8 production [15]. Low molecular weight mushroom glucan (*M*_W_, 5.2 kDa) exhibited effective antioxidant activities in free radicals scavenging and Fe^2+^ chelating [16].

β-Glucan, from *Saccharomyces cerevisiae*, the cell wall component is made up of β-1, 3 linked glucose polymer with varying degree of β-1,6 branching. Yeast β-glucan is an important bioactive compound for animal and human health, but its low solubility has led to many problems. Water-insoluble polysaccharides show little bioactivity, whereas their water-soluble sulfated derivatives exhibit high antitumor and/or antiviral activities [17]. Because of the insoluble chemical nature of β-glucan, particulate β-glucans are not suitable for many medical applications [18]. Sulfation modification could improve its solubility, decrease its *M*_W_, and change its bioactivities. In the homogenous sulfating of yeast β-glucan, the 125 kDa fraction of the sulfated material accounted for only 1% of the product, and 99% of molecular weight of sulfated glucan was 14.5 kDa [19]. Sulfated yeast glucan could stimulate murine bone marrow proliferation [19], and promote chicken lymphocyte proliferation, enhance antibody titer and improve serum IL-2 and IFN-γ concentrations [20]. Previous studies of sulfated glucan were focused on a kind of *M*_W_, but the relationship of *M*_W_ and biological activities of these polysaccharides has been scarcely investigated. In this study, the effect of sulfated yeast β-glucans with different *M*_W_ on the parameters of antioxidant and immune activities in mice was determined.

## 2. Results and Discussion

### 2.1. Results

#### 2.1.1. Chemical Analysis

**Figure 1:**
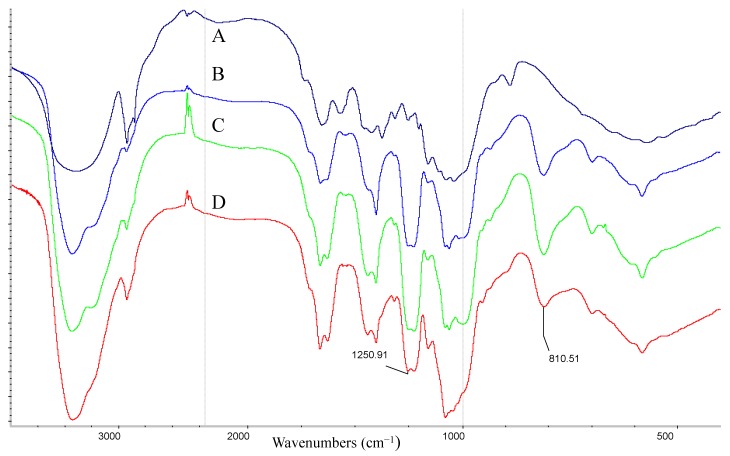
FTIR of GSC (A), sGSC1 (B), sGSC2 (C) and sGSC3 (D).

The *M*_W_ was determined through HPLC-GPC. The *M*_W_ of GSC, sGSC1, sGSC2 and sGSC3 were approximately 896.2, 12.9, 16.5 and 19.2 kDa, respectively. DS was measured using the barium chloride–gelatin method. The DS of sGSC1, sGSC2 and sGSC3 were 0.16, 0.24 and 0.27, respectively. The FTIR of GSC, sGSC1, sGSC2 and sGSC3 in the range of 4000–400 cm^−1^ are shown in Figure 1. Compared with the FTIR spectrum of GSC, those of sGSC1, sGSC2 and sGSC3 showed notable absorption at 1250 and 810 cm^−1^, which corresponded to the S=O stretching and C–O–S vibration of the sulfate ester of hexose. This result indicates that glucan was successfully sulfated.

#### 2.1.2. *In Vitro* Test

##### 1,1-Diphenyl-2-picryl-hydrazyl Radical Scavenging Activity (DPPH RSA)

**Figure 2:**
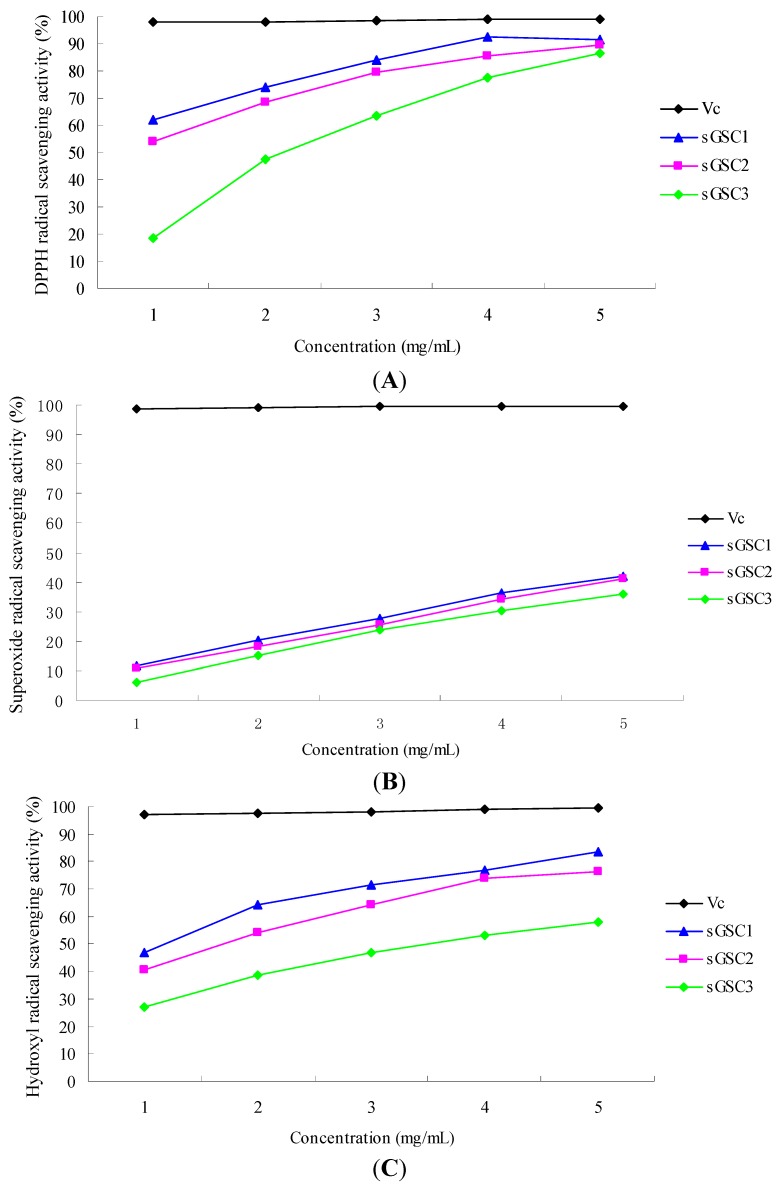
Antioxidant effects of sGSCs: (**A**) DPPH radical scavenging activity; (**B**) Superoxide anion radical scavenging activity; and (**C**) Hydroxyl radical scavenging activity.

Figure 2A shows the DPPH RSAs of the polysaccharide samples and vitamin C (Vc). The scavenging activities increased with increasing concentrations of the sGSCs and Vc. The scavenging rates of V_C_, sGSC1, sGSC2 and sGSC3 at the concentration of 5 mg/mL were 99.00%, 91.59%, 89.72% and 86.56%, respectively. The strength of the DPPH RSAs of the sGSCs was in the order of Vc > sGSC1 > sGSC2 > sGSC3. The results showed that the scavenging abilities of all the sGSCs were lower than that of Vc at each concentration point (*p* < 0.05). However, the scavenging abilities of sGSC1 and sGSC2 were already at a high level.

##### Superoxide RSA

Figure 2B shows the superoxide RSAs of different concentrations of V_C_, sGSC1, sGSC2 and sGSC3. The scavenging activities increased with increasing concentrations of the sGSCs and Vc. The scavenging rates of V_C_, sGSC1, sGSC2 and sGSC3 at the concentration of 5 mg/mL were 99.5%, 42.16%, 41.07%, and 35.72%, respectively. The strength of the superoxide RSAs of the sGSCs was in the order of V_C_ > sGSC1 > sGSC2 > sGSC3.

##### Hydroxyl RSA

Figure 2C shows the hydroxyl RSAs of different concentrations of V_C_, sGSC1, sGSC2 and sGSC3. The scavenging activities increased with increasing concentrations of the sGSCs and Vc. The scavenging rates of V_C_, sGSC1, sGSC2 and sGSC3 at the concentration of 5 mg/mL were 99.50%, 83.41%, 76.12% and 58.14%, respectively. The strength of the hydroxyl RSAs of the sGSCs was in the order of V_C_ > sGSC1 > sGSC2 > sGSC3.

#### 2.1.3. *In Vivo* Test

##### Effect of Glucan Administration on the Activities of Antioxidant Enzymes in Mouse Serum

The effects of the glucans on the activities of superoxidase dismutase (SOD), glutathione peroxidase (GSH-Px), malondialdehyde (MDA) and catalase (CAT) in mouse serum are shown in Table 1. The SOD activities in all glucan groups were not significantly higher than that in the control group (*p* > 0.05). The CAT activities in the sGSC1 and sGSC2 groups were significantly higher than those in the sGSC and control groups (*p* < 0.05). The GSH-Px activity in the sGSC1 group was significantly higher than those in the GSC and control groups (*p* < 0.05). The MDA activities in the sGSC1, sGSC2 and sGSC3 groups were significantly lower than those in the GSC and control groups (*p* < 0.05).

**Table 1:** Effects of glucans on the activities of antioxidant enzymes in mice.

Group	SOD	CAT	GSH-PX	MDA
	(U/mL)	(U/mL)	(μmol/L)	(nmol/mL)
sGSC1	372.54 ± 9.18 ^a^	67.67 ± 1.53 ^c^	449.90 ± 3.61 ^b^	2.03 ± 0.09 ^b^
sGSC2	371.34 ± 7.67 ^a^	56.33 ± 2.51 ^b^	413.20 ± 4.95 ^ab^	2.21 ± 0.04 ^b^
sGSC3	368.66 ± 4.81 ^a^	41.85 ± 1.04 ^a^	378.35 ± 5.44 ^a^	2.38 ± 0.16 ^b^
GSC	371.57 ± 6.07 ^a^	42.00 ± 1.78 ^a^	384.48 ± 7.06 ^a^	5.84 ± 0.22 ^a^
Control group	368.88 ± 3.96 ^a^	37.86 ± 2.83 ^a^	357.30 ± 4.39 ^a^	6.27 ± 0.33 ^a^

Data within a column without the same superscript letters differ significantly (*p* < 0.05).

##### Effect of Glucans on Spleen and Thymus Indexes in Mice

The effects of the glucans on the spleen and thymus indexes in mice are shown in Table 2. Significant differences in the spleen and thymus indexes were detected between all the glucan groups and the control group (*p* < 0.05). These indexes in the sGSC1 group were higher than those in the GSC group (*p* < 0.05), whereas those in the sGSC2 and sGSC3 groups were numerically higher than that in the GSC group (*p* > 0.05).

**Table 2:** Effects of glucans on spleen and thymus indexes in mice.

Group	Spleen Index (%)	Thymus Index (%)
sGSC1	0.32 ± 0.03 ^c^	0.34 ± 0.03 ^c^
sGSC2	0.30 ± 0.02 ^bc^	0.30 ± 0.02 ^bc^
sGSC3	0.29 ± 0.03 ^bc^	0.30 ± 0.01 ^b^
GSC	0.28 ± 0.01 ^b^	0.28 ± 0.02 ^b^
Control group	0.22 ± 0.02 ^a^	0.24 ± 0.03 ^a^

Data within a column without the same superscript letters differ significantly (*p* < 0.05).

##### Effect of Glucan Administration on Spleen Lymphocyte Proliferation in Mice

The effects of the sGSCs on the *A*_570_ values are shown in Table 3. The *A*_570_ values in all glucan groups were significantly higher than those in the control group (*p* < 0.05), whereas those in the sGSC1 and sGSC2 groups were significantly higher than those in the sGSC3 and GSC groups (*p* < 0.05). No significant difference was detected between the sGSC3 and GSC groups (*p* > 0.05).

**Table 3:** Effect of glucans on the spleen lymphocyte proliferation in mice.

Group	*A*_570_ Value
sGSC1	0.72 ± 0.02 ^c^
sGSC2	0.69 ± 0.05 ^c^
sGSC3	0.61 ± 0.04 ^b^
GSC	0.61 ± 0.05 ^b^
Control group	0.50 ± 0.04 ^a^

Data within a column without the same superscript letters differ significantly (*p* < 0.05).

##### Effect of Glucan Administration on CD4^+^and CD8^+^ T Lymphocytes

The effects of glucan on the CD4^+^and CD8^+^ T lymphocytes are shown in Table 4. The percentages of CD4^+^ T cells were significantly higher in the sGSC1, sGSC2, and sGSC3 groups than in the normal control group (*p* < 0.05), whereas these percentages were significantly higher in the sGSC1 and sGSC2 groups than in the GSC group (*p* < 0.05). The percentages of CD8^+^ T cells were significantly lower in the sGSC1, sGSC2, sGSC3, and GSC groups than in the normal control group (*p* < 0.05), whereas these percentages were significantly lower in the sGSC1 and sGSC2 groups than in the GSC group (*p* < 0.05). The ratios of CD4^+^ T cells/CD8^+^ T cells were significantly higher in the sGSC1, sGSC2, and sGSC3 groups than in the normal control group (*p* < 0.05), whereas this ratio was significantly higher in the sGSC1 group than in the GSC group (*p* < 0.05).

**Table 4:** Effects of glucans on spleen lymphocytes in mice.

Group	CD4^+^ (%)	CD8^+^ (%)	CD4^+^/CD8^+^
sGSC1	75.48 ± 0.71 ^d^	20.69 ± 0.37 ^c^	3.77 ± 0.75 ^b^
sGSC2	73.35 ± 0.95 ^c^	21.52 ± 0.18 ^d^	3.41 ± 0.05 ^cd^
sGSC3	71.92 ± 0.82 ^bc^	22.00 ± 0.13 ^bdc^	3.28 ± 0.20 ^d^
GSC	70.74 ± 0.78 ^ab^	23.62 ± 0.50 ^b^	3.00 ± 0.09 ^acd^
Control group	68.99 ± 0.52 ^a^	26.82 ± 0.34 ^a^	2.57 ± 0.10 ^a^

Data within a column without the same superscript letters differ significantly (*p* < 0.05).

##### Effect of Glucan Administration on IL-2 and IFN-γ Secretion in Serum in Mice

The effects of the glucans on the serum concentrations of IL-2 and IFN-γ are shown in Table 5. The serum IL-2 concentrations in the sGSC1, sGSC2, and sGSC3 groups were significantly higher than those in the GSC and control groups (*p* < 0.05), but no significant difference was detected between the GSC and control groups (*p* > 0.05). The serum IFN-γ concentration in the sGSC1 group was significantly higher than those in the GSC and control groups (*p* < 0.05), and the sGSC2 and sGSC3 groups significantly differed from the control group in terms of this parameter (*p* < 0.05).

**Table 5:** Effects of glucans on IL-2 and IFN-γ levels in mice.

Group	IL-2 (pg/mL)	IFN-γ (pg/mL)
sGSC1	85.80 ± 3.56 ^d^	293.16 ± 6.72 ^bc^
sGSC2	75.55 ± 2.21 ^c^	280.69 ± 4.45 ^cd^
sGSC3	66.24 ± 3.31 ^b^	276.55 ± 7.27 ^bcd^
GSC	55.00 ± 4.12 ^a^	268.02 ± 5.96 ^ad^
Control group	54.40 ± 2.30 ^a^	249.80 ± 9.10 ^a^

Data within a column without the same superscript letters differ significantly (*p* < 0.05).

### 2.2. Discussion

The activity of sulfated polysaccharides depends on the *M*_W_, DS, and the position of sulfation [21]. *M*_W_ and DS significantly affects the scavenging ability, but a high *M*_W_ and DS are not necessary to scavenge free radicals [22]. GSC was successfully sulfated, and no notable absorption was observed at 1250 and 810 cm^−1^ corresponding to S=O stretching and C–O–S vibration. Sulfation modification significantly decreased its *M*_W_. The *M*_W_ of GSC was approximately 896.2 kDa, but the sGSC1, sGSC2, and sGSC3 were 12.9, 16.5 and 19.2 kDa, respectively.

Free radicals are continuously produced during metabolism, damage biomolecules, and promote serious health problems [23]. Highly reactive free radicals in biological systems may oxidize nucleic acids, proteins or lipids, and can initiate degenerative disease. ROS, such as superoxide anion, hydroxyl radical, and H_2_O_2_, are generated through normal metabolic processes or from exogenous factors and agents; ROS easily initiate the peroxidation of membrane lipids, leading to the accumulation of lipid peroxides [24]. In the present study, sGSC1, sGSC2, and sGSC3 displayed scavenging effects on DPPH, superoxide anion, and hydroxyl radicals *in vitro*. The activity was in the order of sGSC1 > sGSC2 > sGSC3. The extended chain conformation enhances the biological activity of sulfated polysaccharides [25]. Several defense mechanisms, such as physical, repair, and preventive mechanisms, have been developed to protect against exposure to different free radicals [26]. The antioxidant defense mechanism comprises two components: (1) enzymatic and (2) nonenzymatic. Enzymatic components include CAT, SOD and GSH-Px. MDA is the main product of lipid peroxidation and can reflect the change of oxidative damage and antioxidation. It was nonenzymatic components. Various reviews and research papers have explored the role and mechanism of enzymatic components in protecting against oxidative stress [27,28,29]. The antioxidant enzymes SOD, GSH-Px, and CAT are the first line of antioxidant defense against ROS generated *in vivo* under oxidative stress [28]. In the present study, the oral administration of sGSC1 and sGSC2 notably improved the serum activities of CAT and GSH-Px, and decreased the level of MDA in mice. This finding is consistent with those of previous studies, which indicated that the administration of capsule polysaccharide improves the activities of SOD, GSH-Px, and CAT in a dose-dependent manner [30]. *Ganoderma lucidum* polysaccharides significantly reduce MDA production and increase serum SOD, CAT and GSH-Px activities in cervical carcinoma rats and in ovarian cancer rats [31,32]. Meanwhile, the administration of jujube polysaccharide conjugates and ginsenoside significantly increase SOD and GSH-Px activities and decrease MDA level [33].

The current experiment showed that the strength of the scavenging activities DPPH, superoxide, and hydroxyl radicals was in the order of sGSC1 > sGSC2 > sGSC3 *in vitro*. Moreover, the DS and *M*_W_ of sGSC1, sGSC2 and sGSC3 were 0.16, 12.9 kDa; 0.24, 16.5 kDa; and 0.27, 19.2 kDa, respectively. These results indicate that the scavenging decrease with increasing *M*_W_ and DS *in vitro* and that sGSC1 with low *M*_W_ and DS displays favorable antioxidant activities. The *in vivo* experiment confirmed the results of the *in vitro* experiment. The oral administration of sGSC1 significantly improved serum CAT and GSH-Px activities, and decreased MDA level in mice. This finding agrees with the previous result that sulfated Hunai polysaccharide exhibits a favorable antioxidant capacity and a DS of less than 0.5 [34]. Sulfated lacquer polysaccharide with moderate *M*_W_ and DS has an excellent antioxidant capacity, with a reducing capacity (Fe^3+^–Fe^2+^ transformation) of 0.61 at 500 μg/mL and superoxide and hydroxyl RSAs of 56.4% at 500 μg/mL and 55.6% at 1000 μg/mL, respectively [22].

Natural polysaccharides are important macromolecules that can profoundly affect the immune system and show potential as immunomodulators with broad clinical applications [35]. The spleen and thymus indexes in all glucan groups were significantly higher than those in the control group, and these indexes were higher in the sGSC1, sGSC2 and sGSC3 groups than in the GSC group. This result indicates that the oral administration of both glucan and sulfated glucan can promote the nonspecific immunity of the organism, but sulfation improves the activity of glucan.

The immune response of the body is composed of specific and nonspecific immunity, and the specific immune response includes cellular immunity and humoral immunity [36]. Cellular immunity is mainly mediated by T lymphocytes, which can be proliferated *in vitro* [37]. Lymphocyte proliferation is an important index that reflects cellular immunity and polysaccharide immune-enhancing activity. The present study displayed that the administration of sGSC1 and sGSC2 significantly improved the *A*_570_ values. Thus, these sGSCs can promote lymphocyte proliferation and strengthen cellular immunity.

CD4^+^ and CD8^+^ T cells are key links to immunoregulation in organisms. CD4^+^ T cells contain Th1 and Th2 cells. Th1 activation contributes to cell-mediated immunity, whereas Th2 activation supports the humoral immune response [38]. The Th1/Th2 balance is involved in immunoregulation [39]. Th1 cells induce IFN-γ and IL-2, which trigger the differentiation of CD4^+^ T cells into Th1 cells and inhibit the production of Th2 cells. CD8^+^ T cells are immune effector cells responsible for the removal of target cells through direct killing [40]. In the present study, sGSC1 and sGSC2 effectively improved the percentage of CD4^+^ T cells and decreased the percentages of CD8^+^ T cells as compared with GSC and the normal control. Meanwhile, sGSC1, sGSC2 and sGSC3 significantly affected the CD4^+^/CD8^+^ ratio. Therefore, cellular immune functions were enhanced. Previous studies indicated that *Pyracantha fortuneana* (Maxim.) Li polysaccharides can elevate CD4^+^ T cell count and CD4^+^/CD8^+^ ratio [41].

Cytokines play important roles in the development, function, and control of cells of the immune and other systems. In particular, these potent molecules can influence the differentiation, migration, and proliferation of cells. Cytokine secretion from T lymphocytes plays an important role in immune response. In the present study, the oral administration of sGSC1, sGSC2 and sGSC3 improved serum IL-2 and IFN-γ concentrations in mice. This result indicates that these sGSCs can promote IL-2 and IFN-γ secretion, and thus enhance cellular immunity. This result is also consistent with those of previous studies. For instance, *Inonotus obliquus* polysaccharides can significantly induce the secretion of TNF-α, IFN-γ, IL-1β and IL-2 in peripheral blood mononuclear cells [42]; *Juglan regia* polysaccharides significantly improve the serum levels of immune cytokines (IL-2, TNF-α, and IFN-γ) in tumor-bearing mice [43]; and *Cordyceps militaris* polysaccharides enhance serum IFN-γ and IL-4 concentrations in chicken [37].

In conclusion, the sGSCs improved the immune functions and exhibited effective antioxidant activities *in vitro* and *in vivo*. sGSC1 with low *M*_W_ and DS has better antioxidant and immunological activities than the other sGSCs, which should be explored as a potent antioxidant and immune-enhancing agent.

## 3. Materials and Methods

### 3.1. Reagents

RPMI-1640 was purchased from Gibco (Grand Island, NY, USA). DPPH, Vc, H_2_O_2_, pyrogallic acid, T-cell mitogen Concanavalin A (Con A), bovine serum albumin, and cellulose sacks were purchased from Sigma Chemical Co. (St. Louis, MO, USA). Filter membrane was purchased from Millipore Co. (Billerica, MA, USA). 3-(4,5-Dimethylthia-zol-2-yl)-2,5-dipheny-ltetrazoliumbromide (MTT) was purchased from Amresco Co. Yeast β-1,3-d-glucan standard was purchased from Putus Macromolecular Sci. & Tech. Ltd. (Wuhan, China). Dimethyl sulfoxide (DMSO), phenol, trichloroacetic acid, Coomassie brilliant blue G250, NaOH, acetic acid, urea, and H_2_SO_4_ were purchased from Yixin Institute of Chemical Engineering in Jiangsu. Assay kits for the MDA, CAT, SOD, and GSH-Px were purchased from the Nanjing Jiancheng Bioengineering Institute (Nanjing, China). Anti-CD3-PerCP, anti-CD4-PE, and anti-CD8-FITC antibodies were purchased from BD eBiosciences (San Diego, CA, USA). IL-2 and IFN-γ kits were purchased from R&D systems (Minneapolis, MN, USA). All chemicals used in the experiments were analytical grade.

### 3.2. Preparation of Glucan

Dried *Saccharomyces cerevisiae* was purchased from Zhejiang Shenyou Bio-engineering Corporation, Zhejiang, China. β-Glucan was extracted from *Saccharomyces cerevisiae* using NaOH and acetic acid [43]. In brief, *Saccharomyces cerevisiae* was first sieved (mesh diameter: 80 μm). The solutions were centrifuged at 4500 rpm for 15 min, and then the supernatant was discarded. The sediments were dispersed in a 4% NaOH solution at 90 °C for 2 h and then centrifuged at 3000 rpm for 15 min; the supernatant was decanted. The procedure was repeated twice, and then the sediments were dispersed in deionized water and adjusted to neutral pH. The precipitate was washed twice with ethanol and then lyophilized to obtain GSC. The carbohydrate content of GSC was 78% as measured using the phenol–sulfuric acid method [44].

### 3.3. Preparation of Sulfated Glucan

sGSCs were prepared as described by Williams with some modification [19]. Urea (72 g) was completely dissolved in 100 mL of DMSO and then added with 4 g of GSC. The solution was transferred into a separate flask and then mixed with H_2_SO_4_ and DMSO. The solution was heated to 120 °C in an oil bath with stirring. sGSC1 was added with 10 mL of H_2_SO_4_ mixed into 100 mL of DMSO with stirring for 6 h; sGSC2 was added with 15 mL of H_2_SO_4_ mixed into 100 mL of DMSO with stirring for 2 h; and sGSC3 was added with 15 mL of H_2_SO_4_ mixed into 100 mL of DMSO with stirring for 6 h. After cooling to ambient temperature, the solution was diluted in 1 L of deionized water, filtered to remove impurity [45], ultrafiltered to 100 mL, and then lyophilized. The carbohydrate contents of sGSC1, sGSC2, and sGSC3 were 78.24%, 79.39% and 78.62%, respectively.

### 3.4. Determination of DS

The DS was calculated using the following equation [46]:

(1) $$\text{DS}=\frac{162\times S\%}{32-80\times S\%}$$

where *S*% is the mass ratio of S element in the prepared sGSCs.

### 3.5. Molecular Weight Analysis

The average *M*_W_ was measured through GPC on an ultrahydrogel linear column at 25 °C. The eluent (flow rate: 1.0 mL/min) was a 0.1 M sodium acetate solution. Pullulan standards were used as the standards for *M*_W_ measurement (*M*_W_: 6.1–70.8 kDa).

### 3.6. FT-IR Spectroscopy

The FT-IR spectra of the GSCand sGSCs were obtained and analyzed. Purified polysaccharides (1 mg) and KBr powder (200 mg) were finely ground in an agate mortar and then pressed into thin slices. The FT-IR spectra were obtained in the frequency range of 4000–400 cm^−1^ (Nicolet iS10, Thermo Scientific, Waltham, MA, USA). The major peaks (intensity and wavenumber) were found using OMNIC software (V8.0, Thermo Nicolet, Waltham, MA, USA). The spectra of the polysaccharides were recorded using OMNIC software.

### 3.7. Antioxidant Activities in Vitro

#### 3.7.1. DPPH RSA

The DPPH RSAs of the sGSCs were determined as described by Chen *et al.* [47]. A 1.5 mL aliquot of the sGSC solution (1.0–5.0 mg/mL) was mixed with 1.5 mL of DPPH solution (dissolved in 95% ethanol, 0.1 mmol/L) in tubes. The mixture was shaken and stored in the dark for 30 min at room temperature. The absorbance was obtained at 517 nm. Vc was used as a positive control. The DPPH RSA was calculated using the following equation:

(2) $$\text{DPPH RSA (\%)}=\frac{A_{c}-(A_{i}-A_{j})}{A_{c}}\times 100$$

where *A_c_* is the absorbance value of the DPPH solution without the sGSC solution, *A_i_* is the absorbance of the sGSC solution with the DPPH solution, and *A_j_* is the absorbance of the sGSC solution without the DPPH solution.

#### 3.7.2. Superoxide RSA

The superoxide RSAs of the sGSCs were measured as described by Robak *et al.* [48]. Before the reaction, 4.5 mL of Tris–HCl buffer (50 mM, pH 8.2), 4.2 mL of deionized water, and 0.4 mL of pyrogallic acid (50 mM) were mixed and incubated at 25 °C for 20 min. Then, each sGSC sample was dissolved in deionized water with different concentrations. The mixture was added with 1 mL of sGSC solution and then quickly shaken. After incubating at 25 °C for 5 min, 1 mL of HCl (8 mM) was dripped into the mixture to terminate the reaction. The absorbance was measured at 325 nm. The superoxide RSA was calculated using the following equation:

(3) $$\text{Superoxide RSA (\%)}=\frac{A_{0}-(A_{1}-A_{2})}{A_{0}}\times 100$$

where *A*_0_ is the absorbance of all the reaction reagents with deionized water instead of the sample solution, *A*_1_ is the absorbance of all the reaction reagents with the sample solution, and *A*_2_ is the absorbance of all the samples with Tris–HCl buffer instead of pyrogallic acid.

#### 3.7.3. Hydroxyl RSA

The hydroxyl RSAs of the sGSCs were measured as described by Li *et al.* [17]. In brief, 0.2 mL of brilliant green solution (0.45 mM), 0.5 mL of FeSO_4_ (0.5 mM), 0.5 mL of H_2_O_2_ (3%, *v*/*v*), and 0.5 mL of the sample solution (1.0–5.0 mg/mL) were incubated in test tubes at 25 °C for 30 min and then centrifuged. The absorbance was obtained at 624 nm. Hydroxyl RSA was calculated using the following equation:

(4) $$\text{Hydroxyl RSA (\%)}=\frac{A_{0}-A_{1}}{A-A_{1}}\times 100$$

where *A*_0_ is the absorbance of all the reaction regents with different concentrations of the sample solution, *A*_1_ is the absorbance of all the reaction regents but with deionized water instead of the sample solution, and *A* is the absorbance of all the reaction regents without the sample solution and Fenton reaction system.

### 3.8. Animal and Experimental Design

Male BALB/c mice (8 weeks old) were purchased from Shanghai Slac Laboratory Animal Center of the Chinese Academy of Sciences (Shanghai, China). The animals were provided with water and mouse chow *ad libitum* and were housed in a rodent facility. All procedures involving animals and their care were approved by the Ethics Committee of the Chinese Academy of Agricultural Sciences. The mice were randomly divided into five groups (consisting of 10 mice each). The sGSC1, sGSC2 and sGSC3 treatment groups received the corresponding sGSC at 50 mg/kg/day. The GSC treatment group (positive control) received 50 mg/kg/day GSC. The normal control group received equal volume of physiological saline solution. All treatments were administered intragastrically for 14 day.

#### 3.8.1. Relative Spleen and Thymus Weight

At 24 h after the last polysaccharide administration, the animals were weighed and then sacrificed via decapitation. Spleens and thymuses were immediately removed and weighed. The spleen and thymus indexes were calculated using the following equation: spleen and thymus indexes = spleen or thymus weight/body × 100.

#### 3.8.2. Biochemical Assay

The blood of the mice was collected in a 1.5 mL centrifuge tube without an anticoagulant for 2 h at 37 °C and then centrifuged at 3000 rpm for 15 min at 4 °C. The serum was collected to measure SOD, CAT, GSH-Px and MDA levels and cytokine concentrations. The test was performed in accordance with the manufacturer’s recommendations.

#### 3.8.3. Lymphocyte Proliferation Assay

Three mice of each group were selected to perform the lymphocyte proliferation assay [49]. The mouse spleens were aseptically removed from the sacrificed mice using scissors and forceps in cold Hanks, gently homogenized, and then passed through a cell strainer to obtain single-cell suspensions. The erythrocytes in the cell mixture were washed via hypo-osmotic hemolysis, and the cells were resuspended to a final density of 3 × 10^6^ mL^−1^ in RPMI 1640 medium supplemented with 10% bovine serum albumin. Then, 1 mL of the spleen cell solution was seeded into a 24-well plate containing 75 μL of Con A, and Con A was replaced with 75 μL of deionized water as the control. The plates were cultured at 37 °C in 5% CO_2_ atmosphere for 3 days. After incubation for 68 h, 0.7 mL of solution was removed, and 0.7 mL of RPMI-1640 and 50 μL of MTT (5 mg/mL) were added to each well and incubated for another 4 h. Then, 1 mL of acid isopropyl alcohol was added into each well and evenly mixed. The content of each well of the 24-well plate was added into three wells of a 96-well plate. The absorbance at 570 nm was obtained on a microplate reader (Thermo Multiskan MK3, Waltham, MA, USA).

#### 3.8.4. Detection of CD4^+^ and CD8^+^ T Lymphocytes

The mouse spleens cells were homogenized, sieved, and then centrifuged to obtain single-cell suspensions. The single cells were counted and adjusted to 8 × 10^6^ mL^−1^. Then, 200 μL of cells were centrifuged at 1500 rpm for 8 min at 4 °C. The cells were resuspended in 100 μL of PBS. Afterward, 2.5 μL of anti-CD3-PerCP, 2.5 μL of anti-CD4-PE, and 1 μL of anti-CD8-FITC were applied to stain the cells. The stained cells were incubated at room temperature for 30 min in the dark, washed with PBS, and then centrifuged at 1500 rpm for 5 min at 4 °C. Then, the cells were resuspended in 500 μL of PBS and analyzed using a flow cytometer (Beckman, FC-500MPL, Boulevard Brea, CA, USA).

#### 3.8.5. Serum IL-2 and IFN-γ Concentration Assay

The concentrations of IL-2 and IFN-γ were assayed using an ELISA kit in accordance with the manufacturer’s instructions (R&D systems, Minneapolis, MN, USA). Mouse serum samples were diluted at 1:4 and then incubated in 96-well microtiter plates.

### 3.9. Statistical Analysis

All data were expressed as mean ± S.D. and analyzed using SPSS for Windows version 17.0 (SPSS Inc., Chicago, IL, USA). Duncan’s multiple range test was used to determine the difference among groups. Statistical significance was considered at *p* < 0.05.
